# 12-Month Outcomes of a Prospective Randomized Trial Investigating Effects of IVIG on Top of rATG Versus rATG Alone in Pre-Sensitized Kidney Transplant Recipients: The INHIBIT Study

**DOI:** 10.3389/ti.2025.14312

**Published:** 2025-05-19

**Authors:** Ondrej Viklicky, Ivan Zahradka, Jan Mares, Janka Slatinska, Alena Parikova, Vojtech Petr, Matej Roder, Katerina Jaklova, Klara Osickova, Libor Janousek, Petra Hruba

**Affiliations:** ^1^ Department of Nephrology, Institute for Clinical and Experimental Medicine, Prague, Czechia; ^2^ Transplantation Laboratory, Institute for Clinical and Experimental Medicine, Prague, Czechia; ^3^ Department of Data Science, Institute for Clinical and Experimental Medicine, Prague, Czechia; ^4^ Department of Immunogenetics, Institute for Clinical and Experimental Medicine, Prague, Czechia; ^5^ Department of Transplantation Surgery, Institute for Clinical and Experimental Medicine, Prague, Czechia

**Keywords:** IVIG, desensitization, induction, HLA-incompatible transplantation, kidney transplantation

## Abstract

Intravenous immunoglobulins (IVIG) are commonly used in peri-transplant desensitization, but evidence supporting their efficacy is limited. We conducted a prospective, randomized single-center, open-label, Phase IIIb non-inferiority clinical pilot trial to compare the efficacy of IVIG (administered at a dose of 3 × 0.5 g/kg) versus no IVIG, in conjunction with rabbit anti-thymocyte globulin (5–7 mg/kg) induction, in pre-sensitized patients with donor-specific antibodies who had negative pre-transplantation Flow- and CDC-crossmatches, between July 2020 and November 2022. The primary endpoint was the rate of efficacy failure, defined as biopsy-proven rejection within 12-month post-transplant. Secondary endpoints included the incidence of rejection at protocol biopsies, evaluated by histology and biopsy-based transcripts diagnostics. Of the screened patients, 53 (72.6%) were excluded due to crossmatch positivity. Ten patients were randomized to the IVIG+, and 7 to the IVIG-arm. The trial was prematurely terminated due to futility at interim analysis. In the IVIG-arm, 3 patients (43%) experienced the primary endpoint compared to none in the IVIG+ arm (p = 0.026). MMDx identified one molecular ABMR in the IVIG+ and 2 in the IVIG-arm in 12-month protocol biopsies. There was one graft loss in the IVIG-arm. The results of this pilot study, although not definitive, do not support the use of IVIG-sparing regimens in HLA-incompatible kidney transplantation (NCT04302805).

This study is registered on ClinicalTrials.gov under the identifier NCT04302805.

## Graphical Abstract

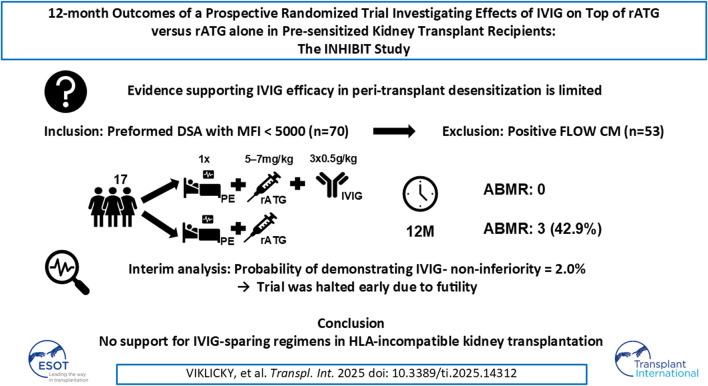

## Introduction

Kidney transplantation across the HLA barrier is associated with an increased risk of antibody-mediated rejection (ABMR) and inferior transplantation outcomes [1, 2]. Consequently, the presence of donor specific anti-HLA antibodies (DSA) prior to transplantation is often met with reluctance to accept transplant offers. However, a too cautious approach is impractical for broadly sensitized patients, who thus often wait for many years and are at risk of never being transplanted. To address this, some centres offer HLA-incompatible (HLAi) transplantations to highly sensitized patients, after carefully weighing the associated risks and benefits [3, 4].

Desensitization and induction protocols in HLAi transplantation are based mainly on centre experiences rather than robust data-based evidence [5]. Among the desensitization armamentarium are intravenous immunoglobulin (IVIG) that have been widely used for decades [6, 7]. The mechanism of action is not well understood, but several have been proposed, including non-specific blockade of Fc receptor, expansion of regulatory T cells, inhibition of B-cell activation and proliferation, inhibition of antibody rebound or immunoregulatory functions of natural antibodies [8–14]. However, the use of IVIG for desensitization has not yet received FDA approval, and the evidence supporting this practice remains limited and weak [5]. In the sole randomized controlled trial performed to date, graft survival was comparable between IVIG and placebo groups, but higher rejection rate was observed in those treated with IVIG despite a reduction in panel-reactive antibodies (PRA) [8]. Observational studies have suggested potential benefits of IVIG, however, these studies should be interpreted with caution due to their retrospective and observational design [15].

Given the limited supporting data, high cost and limited availability due to the human origin of the products, further research into its role in peri-transplant desensitization is of clinical importance. Therefore, we conducted an investigator-initiated randomized trial to evaluate whether rabbit anti-thymocyte globulin (rATG) alone is as effective as rATG combined with IVIG, which is currently the standard of care in HLAi kidney transplantation.

## Materials and Methods

### Study Design and Population

This is a prospective interventional randomized single-centre open-label two-arm Phase IIIb non-inferiority investigator-initiated pilot clinical trial. The aim was to determine whether the induction with rATG alone (intervention) is clinically non-inferior to rATG combined with IVIG (centre standard of care) in preventing biopsy proven rejection within the first 12 months following HLAi transplantation. This study was approved by the Ethics Committee of the Institute for Clinical and Experimental Medicine and Thomayer Hospital (No. A-19-13) and was conducted in accordance with the Helsinki Declaration and good clinical practice guidelines. The clinical trial is registered at ClinicalTrials.gov under Identifier NCT04302805.

The main inclusion criterion was the presence of low levels of preformed anti-HLA DSA, defined as mean fluorescence intensity (MFI) of <5,000 (class I and class II antibodies, except for DQ antibodies where higher MFI values might be accepted). The main exclusion criterion was flow-cytometry crossmatch (FCXM) and/or complement-dependent cytotoxic (CDC) crossmatch positivity prior to transplant surgery. Therefore, the study population consists of patients at category 3 risk of current recommendations of the ENGAGE working group [4]. All the inclusion and exclusion criteria are listed in Supplementary Table S1.

Participants were enrolled at the study centre by the study investigators. Participants were sequentially allocated a unique identification number that was generated electronically via an electronic case report form by the study investigators. Participants were randomized either into the IVIG- or the IVIG+ group prior to the transplantation by a stratified randomization algorithm and assigned to intervention by the study investigators. Random allocation was made in blocks of 4 in a 1:1 ratio and was stratified according to baseline characteristics: gender (male vs. female), donor type (deceased vs. living donor) and type of transplantation (first transplantation vs re-transplantation). The planned follow-up period was 12 months, with protocol biopsies scheduled at months 3 and 12. The study visit schedule and procedures are outlined in Supplementary Table S2.

### Treatment Protocol

After obtaining written informed consent from participants, all patients underwent a single plasma exchange (1x total plasma volume) immediately before kidney transplantation. IVIG was administered at post-operative days (POD) 1, 3, and 5 at a dose of 0.5 g/kg [15]. Rabbit anti-thymocyte globulin (rATG; Thymoglobulin, Sanofi) was initially administered during transplant surgery (1.5 mg/kg), followed by daily doses until a cumulative dose of between 5 mg/kg and 7 mg/kg was achieved. Methylprednisolone 500 mg was given prior to reperfusion and on POD1. Maintenance immunosuppression consisted of once-daily extended-release tacrolimus formulation given pre-transplantation with target range of 8–12 ng/mL, mycophenolate mofetil (2000 mg tapered to 1,000 mg by month 3) and tapered prednisone, starting at 20 mg.

Infection prophylaxis consisted of valganciclovir for 100 days and trimethoprim/sulfamethoxazole 480 mg/day for 6 months. Further details regarding the study treatment protocol can be found in Supplementary Table S3.

### Outcome Measures

The primary outcome measure was efficacy failure, defined as biopsy-proven ABMR and/or T-cell mediated rejection (TCMR) according to the Banff 2017 classification regardless of biopsy indication (for-cause or per-protocol) up to 12 months post-transplantation.

Secondary efficacy outcomes included the incidence of individual rejection types and biopsy findings (active ABMR, chronic active ABMR, acute TCMR, chronic TCMR) both in for cause and protocol biopsies, time to active ABMR, and incidence of delayed graft function (DGF). Molecular assessment of all available 12-month protocol biopsies was conducted using the Molecular Microscope Diagnostic System (MMDx) platform [16]. The dynamics of estimated glomerular filtration rate (eGFR), albuminuria (expressed as albumin/creatinine ratio; ACR) and donor-specific antibodies (DSA) were evaluated at regular time-points.

Secondary safety outcomes included the incidence of all-cause mortality, graft loss, leucopenia (requiring treatment of immunosuppression adjustment), post-transplant diabetes mellitus (PTDM), cardiovascular disease, malignancy and infectious complications, including bacterial infections and viral infections such as BK polyomavirus (BKV) and cytomegalovirus (CMV).

### Anti-HLA Antibody Evaluation

Anti-HLA antibodies were analysed using single antigen bead (SAB) technology with LabScreen Mixed and LabScreen Single Antigen Luminex technique (both One Lambda, Inc.). Donor HLA typing was used for the assessment of donor specific antibodies (DSA). Organ donors were typed using polymerase chain reaction sequence specific primer (SSP) low-resolution kits (Olerup SSP, and Histo Type SSP, BAG). DSA assessment was performed with the HLA fusion software (One Lambda, Inc.). FCXM was performed according to previously described methodology [17].

### Sample Size Calculation

The primary hypothesis of clinical non-inferiority of IVIG-as compared to IVIG+ at the non-inferiority margin of 20% (absolute incidence) of the primary endpoint was chosen to be tested by a one-sided 90% Wald confidence interval for difference in incidence rates. Based on the assumption of expected incidence rates of 45% in both study groups, the required sample size for 80% study power was calculated to be 138 patients total (69 per study group) while correcting for 20% drop out. The reduced level of statistical significance and the relatively large non-inferiority margin were chosen with regard to the limited number of potential participants available in the population and also to the fact that IVIG+ was the centres’ standard of care. Therefore, it was considered more ethical to first conduct a study with smaller sample size to limit the exposition of a potentially inferior treatment to many patients, despite the limited conclusion that could have been made due to increased chance of the type I error.

### Statistics

Statistical analysis was performed using R, version 4.3.2 (R Core Team 2023; R: A Language and Environment for Statistical Computing. R Foundation for Statistical Computing, Vienna, Austria. URL^1^).

Continuous variables are reported as medians with interquartile ranges (IQR), categorical as proportions (%). Barnard’s test, an alternative to the Fisher’s test that is suited for examining contingency tables with a single fixed marginal (as in this study), was used to compare categorical variables, including primary and secondary outcomes. The non-parametric Wilcoxon test was used to compare continuous variables. In the interim analysis, the conditional power was evaluated by performing random simulations of the trial with the expected event rates given as equally weighted averages of the observed rates and the originally assumed rates. Following the early termination of the study at the interim analysis (described below) due to futility in demonstrating the non-inferiority of IVIG-, a post-hoc test for differences in the main outcome between the study groups was conducted. Confidence intervals for the primary outcome were calculated using the Miettinen-Nurminen method. The alpha level for this post-hoc test and for all the other analyses performed was the common standard of 5%. The full statistical analysis plan is provided in Supplementary Material S1.

## Results

### Patient Characteristics

**FIGURE 1:**
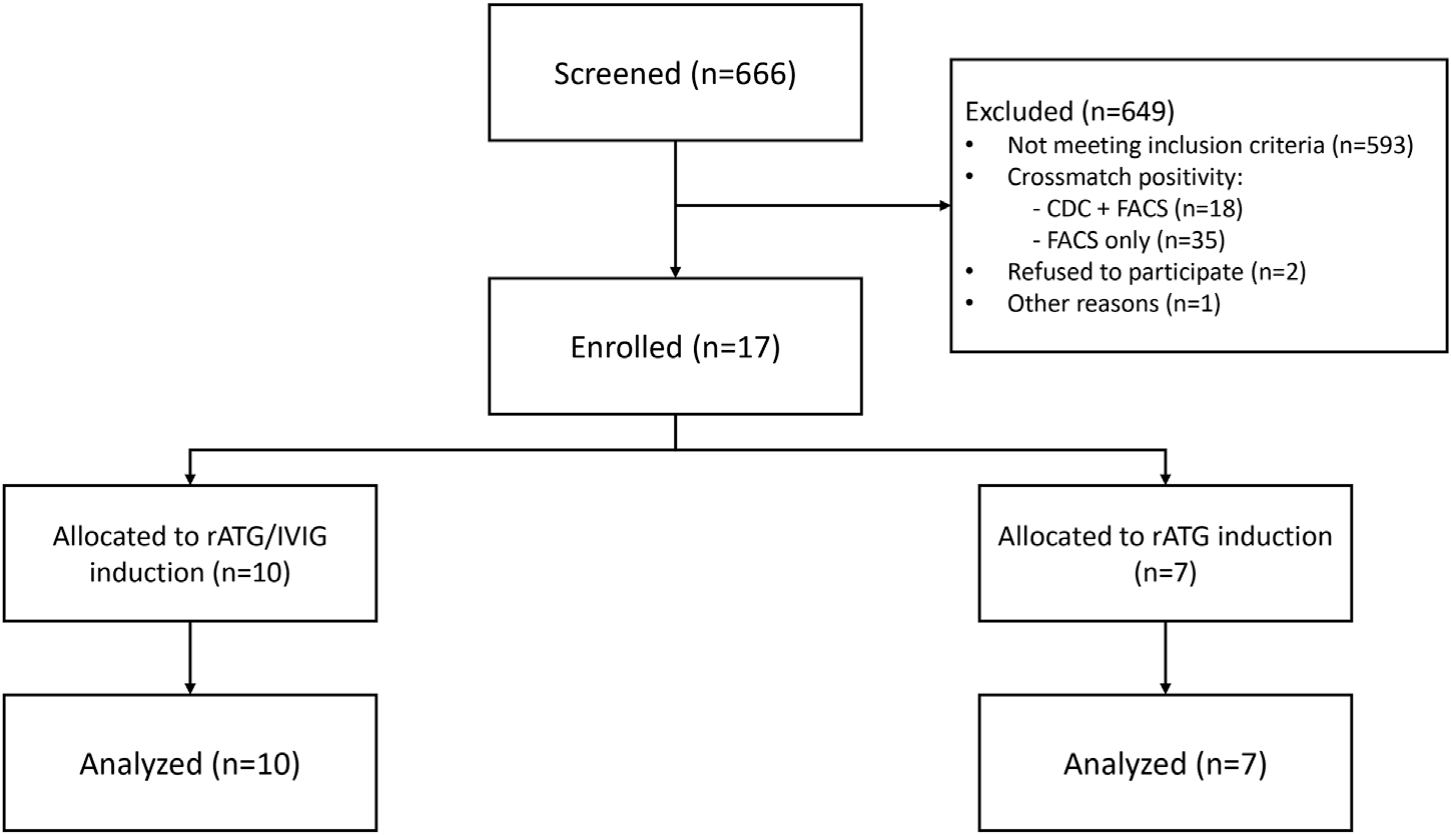
Study flow chart. A total of 666 patients were screened during the study period; 593 did not meet the inclusion criteria, mainly by the absence of donor specific antibodies (DSA). From the 73 DSA positive patients who met the other inclusion criteria, 56 could not be enrolled due to CDC and/or FCXM crossmatch positivity. Finally, 17 patients undergoing HLA-incompatible transplantation were enrolled, 10 were randomized into the rATG/IVIG (IVIG+) group, and 7 were randomized into the rATG without IVIG (IVIG-) group.

A total of 17 patients were randomized between 18 September 2020, and 23 November 2022, with 10 assigned to the IVIG+ group and 7 to the IVIG-group. Baseline characteristics are shown in Table 1, and the study flowchart is presented in Figure 1.

**TABLE 1:** Study population characteristics.

	IVIG+ (n = 10)	IVIG- (n = 7)
Sex (male), n (%)	5 (50%)	2 (28.6%)
Age (years), median (IQR)	58 (44.8–60.9)	53.4 (45.1–58.6)
CPRA (%), median (IQR)	96.4 (69.2–99.1)	66.9 (48.3–77.1)
PRA max (%), median (IQR)	14 (11.5–55)	22 (12–37)
HLA mismatch, median (IQR)	3 (2–5)	4 (4–6)
Re-transplantation, n (%)	5 (50%)	3 (42.9%)
CMV mismatch, n (%)	3 (30%)	1 (14.3%)
Donor age (years), median (IQR)	48 (39.5–56.8)	52 (42–57)
Dialysis vignette duration (years), median (IQR)	2.4 (0.6–3.8)	4.3 (1.1–4.6)
Deceased donor, n (%)	10 (100%)	7 (100%)

Abbreviations: CMV, cytomegalovirus; cPRA, calculated panel reactive antibodies; HLA, human leukocyte antigen; PRA, panel reactive antibodies; IQR, interquartile range.

### Interim Analysis and Trial Termination

**FIGURE 2:**
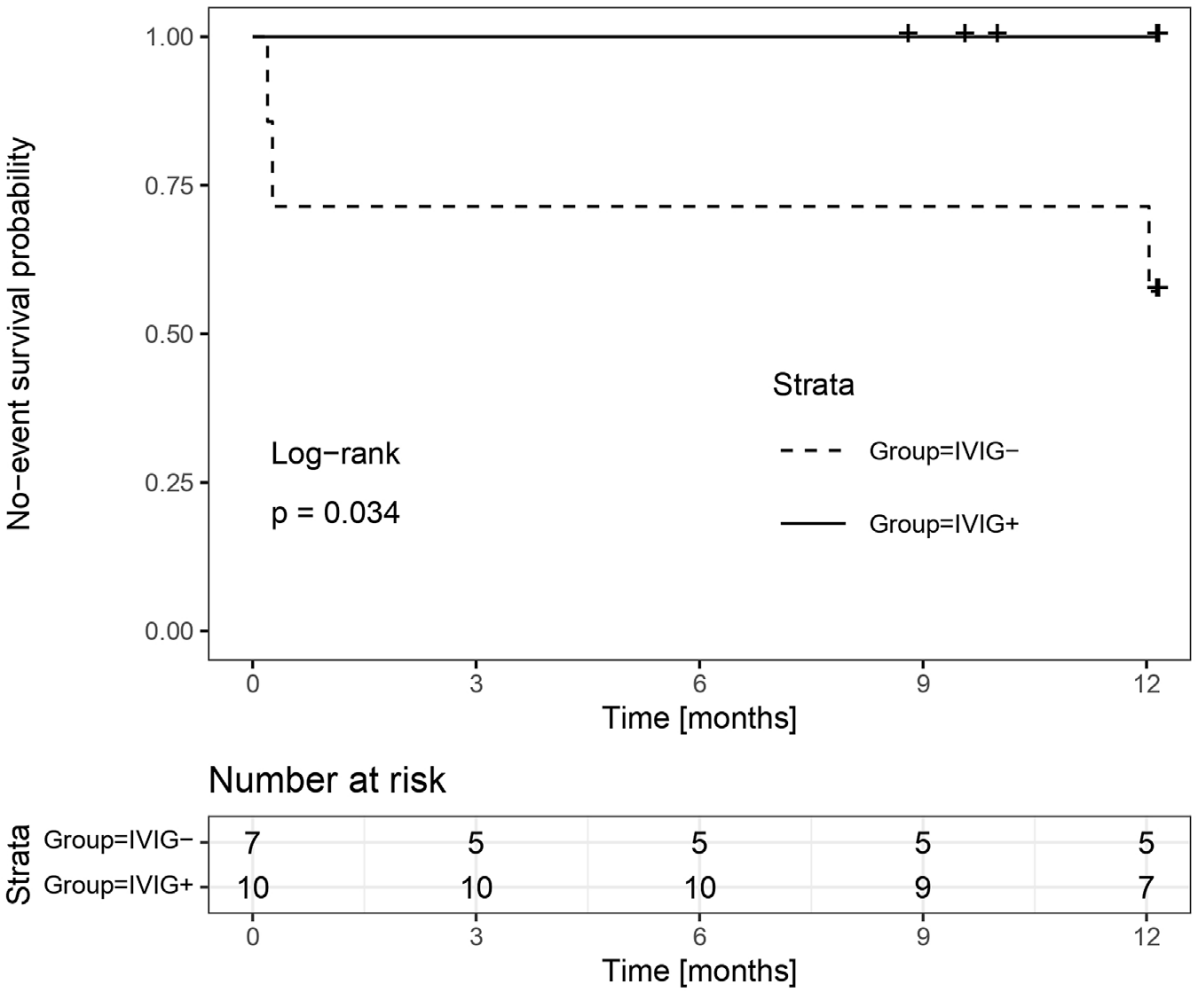
Interim analysis. Rates of the primary outcome at the time of the interim analysis estimated with the use of the Kaplan-Meier method. The primary outcome measure was efficacy failure defined as a biopsy proven antibody mediated rejection and/or T-cell mediated rejection up to 12 months post-transplantation.

An interim analysis was conducted earlier than originally planned due to slower-than-expected enrolment. At this point, 17 patients had been enrolled. It was revealed that at the time of the interim analysis there were 3 primary events in the IVIG-group, while no primary event occurred in the IVIG+ group (Figure 2). The one-sided 90% confidence interval (CI) for the difference in event rates based on these data was (-∞, +83%), far exceeding the non-inferiority margin of 20%. Moreover, the conditional power (the probability of demonstrating IVIG- non-inferiority if the study continued until the originally planned sample size after updating assumptions about the future based on the data that was already observed) was only 2.0%. Given these findings, the trial was terminated for futility.

### Primary Endpoint and Incidence of Antibody-Mediated Rejection

After the 1-year follow-up, the primary endpoint occurred in 3 patients (42.9%) in the IVIG-arm and 0 patients (0%) in the IVIG+ arm (p = 0.026), with a one sided 90% CI of −100%–66.4%, which is not in favour of non-inferiority of IVIG-at the non-inferiority margin of 20% absolute difference. The corresponding two-sided 95% CI was 6.8%–75.6%, indicating the superiority of IVIG+ over IVIG-.

Of the three primary endpoint occurrences, there were two cases of active ABMR early after transplantation diagnosed at post-operative day 6 and 8, respectively, and one case of chronic active ABMR at a 12-month protocol biopsy. There were no instances of acute or chronic T-cell mediated rejection during the study period.

There was one case of graft loss in the IVIG-arm on day 8 post-transplantation. This was due to a case of ABMR which presented with TMA and extensive infarctions at the time of biopsy. Importantly, CDC crossmatch was performed on the day of biopsy, which was positive. Graftectomy was performed due to the serious histology finding and poor prognosis.

### Protocol Biopsies at 3 and 12 Months

Protocol biopsies were performed in 14 out of 16 patients (87.5%) with functioning grafts at 3 months and 13 out of 16 patients (81.3%) at 12 months, with 2 and 3 patients, respectively, declining the biopsy and 1 experiencing graft loss prior to the 3-month mark.

Histological evaluation of the 3-month biopsies revealed no definitive rejections while the MMDx assessments were not performed at this time-point. The evaluation of the 12-month biopsies revealed one case of chronic active ABMR in the IVIG-arm, confirmed by biopsy-based transcripts assessment evaluated by the Molecular Microscope Diagnostic System (MMDx). MMDx also identified early-stage of molecular ABMR in 2 additional biopsies, one from each arm, that did not fully satisfy the Banff criteria for ABMR (Supplementary Table S4).

Molecular rejections were not counted towards the primary endpoint, as molecular assessment was not originally planned for in the study protocol as the MMDx platform was not available at the study’s inception and it was not performed in all biopsies. However, a post-hoc analysis, incorporating both histological and molecular findings, identified 3 events (43%) in the IVIG-group and 1 event (10%) in the IVIG+ group (p = 0.16).

### Evolution of Anti-HLA Donor-Specific Antibodies During the Study Period

**FIGURE 3:**
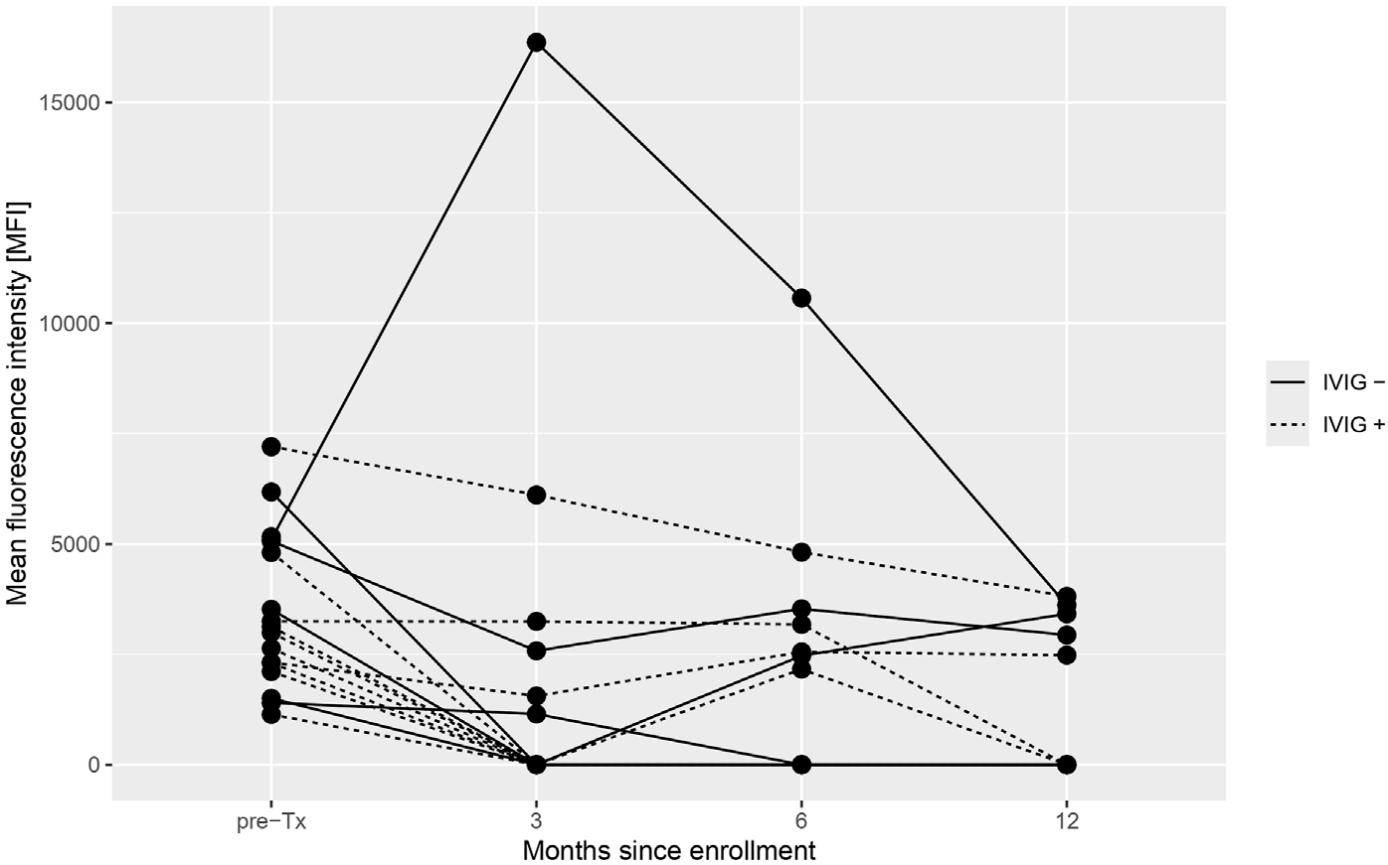
Development of immunodominant donor-specific antibodies (DSA) of each enrolled patient during the 12-month study period DSA were measured during the predefined timepoints at month 3, 6, and 12. Immunodominant DSA was defined as the DSA with the highest mean fluorescent intensity prior to transplantation.

In 11 patients (64.7%), pre-formed DSA either decreased or became undetectable during follow-up, with only one patient in the IVIG- group showing notable transient increase in preformed DSA. One case of *de-novo* DSA with low MFI was observed in the IVIG+ group. The dynamics of immunodominant DSA for each patient during follow-up are shown in Figure 3 and detailed in Supplementary Table S5.

### Safety Outcomes

No patients died during the study follow-up. The incidence of delayed graft function did not differ significantly between the IVIG+ and IVIG-groups (42.9% vs. 30%, respectively; p = 0.62). Similarly, there were no significant differences in the frequency of bacterial or viral infections between the groups. For a comprehensive overview of the secondary outcomes see Table 2. Details of therapeutic drug monitoring are provided in Supplementary Table S6.

**TABLE 2:** Secondary and safety outcomes.

	IVIG+ (n = 10)	IVIG- (n = 7)	p-value
Secondary efficacy outcomes			
Incidence of active ABMR, n (%)	0 (0%)	2 (28.6%)	0.077
Incidence of chronic active ABMR, n (%)	0 (0%)	1 (14.3%)	0.26
Incidence of acute or chronic TCMR, n (%)	0 (0%)	0 (0%)	1
Delayed graft function, n (%)	3 (42.9%)	3 (30%)	0.62
eGFR at 12 months, mL/min/1.73 m^2^ (IQR)	0.89 (0.62–1.05)	1.33 (0.72–1.41)	0.32
Albumin/creatinine ratio at 12 months, g/mol (IQR)	2.3 (1.9–3.5)	4.2 (0.6–18.2)	0.64
Secondary safety outcomes			
Mortality during the study period, n (%)	0 (0%)	0 (0%)	1
Graft loss during the study period, n (%)	0 (0%)	1 (14.3%)	0.26
Leucopenia requiring treatment or immunosuppression adjustment, n (%)	4 (40%)	3 (42.9%)	0.92
Incidence of cardiovascular disease, n (%)	3 (30%)	0 (0%)	0.16
Incidence of post-transplant diabetes mellitus, n (%)	1 (10%)	0 (0%)	0.77
Incidence of malignancy, n (%)	0 (0%)	1 (14.3%)	0.56
Bacterial infection requiring antibiotic therapy, n (%)	7 (70%)	4 (57.1%)	0.6
COVID-19, n (%)	2 (20%)	4 (57.1%)	0.13
CMV replication above 1,000 copies/mL or CMV disease, n (%)	2 (20%)	1 (14.3%)	0.9
EBV replication above 500 copies/mL, n (%)	1 (10%)	1 (14.3%)	0.98
BKV replication above 10,000 copies/mL or BKV nephropathy, n (%)	0 (0%)	0 (0%)	1
Permanent discontinuation of study treatment, n (%)	0 (0%)	0 (0%)	1

Abbreviations: ABMR, antibody mediated rejection; BKV, BK polyomavirus; CMV, cytomegalovirus; COVID-19, coronavirus disease 2019; eGFR, estimated glomerular filtration rate; EBV, Eppstein-Barr virus; IQR, interquartile range; TCMR, T-cell mediated rejection.

## Discussion

The use of IVIG in peri-transplant desensitization protocols for HLA-incompatible kidney transplantation is widespread, despite the low quality of supporting evidence [5]. In this prospective, randomized trial, we hypothesized that an IVIG-free induction protocol would be as efficacious as a combined regime, provided the immunological risk was well characterized at the time of transplantation. However, the findings from the pilot INHIBIT study, although not definitive, do not support the use of IVIG-sparing regimens in HLA-incompatible kidney transplantation, even when pre-transplant FCXM is negative, and DSA levels are low.

Recently, several groups have studied outcomes of DSA positive kidney transplantation, demonstrating a high incidence of ABMR, including subclinical cases [3, 18–20]. Outcomes of HLAi transplantation depend on how pretransplant risks are defined. The definition of acceptable DSA levels for transplantation remains unclear and varies considerably across transplant centres. Some centres do not accept DSAs of any level, in other centres certain DSA levels are acceptable when CDC crossmatch is negative and peri-transplant desensitization is applied. Moreover, DSA MFI thresholds for organ acceptance also vary, influenced by centre practice, analytical platform, antigen type, and delisting strategies among others [4, 21–25]. One approach adopted by several centres, including ours, is to accept HLAi kidney offers if the FCXM is negative, as the risk of ABMR is arguably acceptable [17, 26, 27]. In our study, however, the inclusion of FCXM testing for all HLA-incompatible transplants was associated with a 76% FCXM positivity among “low” DSA positive patients who were invited to the centre for a physical crossmatch. While this approach likely improved the identification of patients at lower risk of ABMR, it also contributed to an unexpectedly high drop-out rate and delayed enrolment. However, the rates of ABMR were much higher when FCXM was not available for decision making prior to HLAi transplantations in our centre in the past [17], and similarly, high ABMR occurrences were observed in several previous observational studies where FCXM-positive patients were transplanted [28–30].

Importantly, our study used an approximate MFI threshold of <5,000 as one of the criteria for inclusion. While some studies indicate that patients with preformed DSA in the range of 5,000–10,000 MFI may have similar outcomes to those with MFI <5,000 [20], others suggest that MFI >5,000 is associated with a substantially increased risk of ABMR [31]. Given the intervention in our study was IVIG elimination, we opted for a more conservative DSA MFI threshold and implemented FCXM pretransplant as a go/no go rule. Therefore, our study population corresponds to category 3 of the recent ENGAGE recommendations, characterized by acceptable medium-term graft survival, but with recommended adaptation of immunosuppression [4]. IVIG is frequently employed in such cases, and our study provides further evidence supporting its utility.

Molecular diagnostic methods are currently being recommended to improve diagnostic precision in HLAi transplantation [32]. To better understand intragraft molecular processes, we retrospectively performed biopsy-based transcripts diagnostics using validated MMDx platform in all available 12-month protocol biopsies. Molecular rejection was identified in two cases in the IVIG-sparing arm and one case in the IVIG+ arm, with only one of these cases showing clear corresponding histological finings. These results suggest that molecular diagnostics may offer a sensitive tool to clarify ABMR cases in biopsies with histologic ambiguity [18, 33–36].

Encouragingly, no rejection episodes by histology occurred in the IVIG+ group during follow-up. The explanation why IVIG therapy given early after transplantation might be effective likely stems from its proposed desensitization mechanism of action. In most patients, DSA levels decreased and remained below the threshold of positivity during follow-up, while persistent or increasing DSA levels were observed in all ABMR cases, all of whom were in the IVIG-group. However, as not all patients with persistent DSA developed ABMR, be it histological or molecular, it is possible that other immune mechanisms, such as those involving plasma cells or natural killer cells, contribute to ABMR pathogenesis, as is recently discussed [33].

A major limitation of our study was the lower-than-expected sample size, which was due to the slow enrolment rate associated with frequent FCXM positivity among DSA-positive patients. Nonetheless, the study was ultimately terminated early for futility, as interim results indicated that proving non-inferiority of the IVIG-sparing regimen was highly unlikely, even if the planned number of participants had been reached. Contrary to our hypothesis, significantly higher rates of the primary endpoint were observed in the IVIG-sparing arm, and this important biological signal must be taken seriously. Furthermore, in theory, type I error could have been inflated if study results were tested multiple times during enrolment and the trial terminated whenever a significant result would have been reached. However, in the case of our study, the data were analysed only at a single time point during the interim analysis so the risk of type I error should not be increased.

In conclusion, the results of this pilot study, although not definitive, do not support the use of IVIG-sparing regimens in HLA-incompatible kidney transplantation, despite the low number of participants and premature study termination. IVIG, the current standard of care, should likely remain an integral part of induction protocols to achieve the best possible outcomes in patients undergoing HLA-incompatible transplantation.

## Data Availability

The raw data supporting the conclusions of this article will be made available by the authors, without undue reservation.

## Footnotes

1
https://www.r-project-org

## Abbreviations

ABMR, antibody-mediated rejection; ACR, albumin/creatinine ratio; BKV, BK polyomavirus; CDC, complement-dependent cytotoxic crossmatch; CMV, CI cytomegalovirus confidence interval; cPRA, calculated panel-reactive antibodies; DGF, delayed graft function; DSA, donor specific antibodies; EBV, Eppstein-Barr virus; eGFR, estimated glomerular filtration rate; FCXM, flow-cytometry crossmatch; HLA, human leukocyte antigen; IQR, interquartile ranges; IVIG, Intravenous immunoglobulins; KTR, kidney transplant recipient; MFI, mean fluorescence intensity; MMDX, Molecular Microscope Diagnostic System; POD, post-operative day; PRA, panel-reactive antibodies; PTDM, post-transplantation diabetes mellitus; rATG, rabbit anti-thymocyte globulin; SAB, single antigen bead; SSP, sequence specific primer; TCMR, T-cell mediated rejection.
