# Supplementary material for: 12-Month Outcomes of a Prospective Randomized Trial Investigating Effects of IVIG on Top of rATG Versus rATG Alone in Pre-Sensitized Kidney Transplant Recipients: The INHIBIT Study
Source: Transpl Int. 2025 May 19;38:14312. doi: 10.3389/ti.2025.14312 (PMC12127847; doi:10.3389/ti.2025.14312)
Supplement: Supplementary file 1 [file DataSheet1.docx]

**Table of contents**

[Supplementary Table 1: Inclusion and exclusion criteria 1](#_Toc560947359)

[Supplementary Table 2: Study procedures 2](#_Toc542203532)

[Supplementary Table 3. Study medication protocol 3](#_Toc1335306215)

[Supplementary Table 4: Histological Banff categories and Molecular Microscope (MMDx) in patients with rejection 4](#_Toc1868671846)

[Supplementary Table 5. Development of donor-specific antibodies of each enrolled patient during the 12-month study period 5](#_Toc847255240)

[Supplementary Table 6. Therapeutic drug monitoring 6](#_Toc634460944)

[Supplementary Material 1. Statistical analysis plan 7](#_Toc7255432)

[Supplementary Material 2. Capsule Sentence Summary 10](#_Toc1249086237)

# **Supplementary Table 1:** Inclusion and exclusion criteria

| **Inclusion criteria:** |
| --- |
| 1. Primary deceased donor or living donor kidney transplantation (first transplantation or re-transplantation) |
| 2. Recipient age ≥ 18 years and < 70 years |
| 3. Donor age < 70 years |
| 4. Written Informed Consent and Consent for Processing Personal Data |
| 5. Last anti-HLA screening no longer than 12 months with positive results |
| 6. MFI of class I or II DSA in the range of 1 000 – 5 000 with the exception of MFI DSA 1 000-15 000 for anti DQ when available at randomization. |
| **Exclusion Criteria:** |
| 1. Combined kidney transplantation with another organ |
| 2. Immunosuppressive therapy up to 6 months before transplantation |
| 3. AB0 incompatible transplantation |
| 4. Women in childbearing potential without adequate contraception |
| 5. HIV positivity |
| 6. Leukopenia < 3 000, thrombocytopenia < 75 000 |
| 7. Tuberculosis history |
| 8. Anti-HCV positivity, HBsAg positivity or HBV DNA positivity |
| 9. DSA measured by Luminex with MFI > 5 000 known at screening prior to transplant, anti DQ ˃15 000 if known. |
| 10. FACS T and B CM positivity known at screening prior to transplant |
| 11. Positive CDC prior to transplantation |
| 12. Planned PP/PE and RTX treatment post-transplant |
| 13. Advanced liver disease (Child-Pugh C or laboratory values of ALT or AST more than 3 times upper limit of normal range) |
| 14. Pregnancy, breastfeeding |
| 15. Study medication is contraindicated according to the Summary of product characteristics |
| 16. Patient is enrolled in other clinical trial |

# **Supplementary Table 2:** Study procedures

| **Procedure** | **D0** | **D7** | **D14** | **M1** | **M2** | **M3** | **M6** | **M12** |
| --- | --- | --- | --- | --- | --- | --- | --- | --- |
| Physical examination | x | x | x | x | x | x | x | x |
| Vital signs | x | x | x | x | x | x | x | x |
| Biopsy |  |  |  |  |  | x |  | x |
| Laboratory – biochemistry: Cr, eGFR, Ualb/CR, proteinuria | x | x | x | x | x | x | x | x |
| Laboratory – biochemistry: ALT, AST | x |  |  |  |  | x | x | x |
| Blood cells count | x | x | x | x | x | x | x | x |
| LUMINEX (DSA, de novo DSA) | x |  |  |  |  | x | x | x |
| Pregnancy test | x |  |  |  |  | x | x | x |
| CMV, EBV, BKV viral load | x |  |  |  | x | x | x | x |
| FACS CM | x |  |  |  |  |  |  |  |

Abbreviations: BKV, BK polyomavirus; CMV, cytomegalovirus; Cr, creatinine; COVID-19, coronavirus disease 2019; eGFR, DSA, donor-specific antibodies; estimated glomerular filtration rate; EBV, Eppstein-Barr virus; FACS CM, flow-cytometry crossmatch

# **Supplementary Table 3.** Study medication protocol

| **Medication** | **POD 0** | **POD 1** | **POD 2** | **POD 3** | **POD 4** | **POD 5** | **POD 6** | **POD 7** | **POD 14** | **M 1** | **M 2** | **M 3** | **M 6** | **M 12** |
| --- | --- | --- | --- | --- | --- | --- | --- | --- | --- | --- | --- | --- | --- | --- |
|  | **baseline** |  |  |  |  |  |  | **Visit 1** | **Visit 2** | **Visit 3** | **Visit 4** | **Visit 5** | **Visit 6** | **Visit 7** |
| rATG | 1.5 mg/kg | 1 mg/kg | 1 mg/kg | 1 mg/kg | Until 5-7 mg/kg total | - | - | - | - | - | - | - | - | - |
| IVIG | - | 0.5 g/kg | - | 0.5 g/kg | - | 0.5 g/kg | - | - | - | - | - | - | - | - |
| Plasma exchange | 1 | - | - | - | - | - | - | - | - | - | - | - | - | - |
| Tacrolimus | 0.17 mg/kg | 0.17 mg/kg | 10-15 ng/ml | 10-15 ng/ml | 10-15 ng/ml | 10-15 ng/ml | 10-15 ng/ml | 10-15 ng/ml | 10-15 ng/ml | 7-12 ng/ml | 5-8 ng/ml | 5-8 ng/ml | 5-8 ng/ml | 5-8 ng/ml |
| Methylprednisolone | 500 mg | 500 mg | - | - | - | - | - | - | - | - | - | - | - | - |
| Prednisone | - | - | 20 mg | 20 mg | 20 mg | 20 mg | 20 mg | 20 mg | 15 mg | 10 mg | 5 mg | 5 mg | 5 mg | 5 mg |
| Valganciclovir | + | + | + | + | + | + | + | + | + | + | + | + | until day 100 | - |
| Cotrimoxasol (Trimethoprim/ Sulfamethoxazole) | 480 mg | 480 mg | 480 mg | 480 mg | 480 mg | 480 mg | 480 mg | 480 mg | 480 mg | 480 mg | 480 mg | 480 mg | 480 mg | - |

Abbreviations: IVIG, intravenous immunoglobulins; POD, post-operative day; rATG, rabbit anti-thymocyte globulin

# **Supplementary Table 4:** Histological Banff categories and Molecular Microscope (MMDx) in patients with rejection

| **Patient** | **Time of biopsy** | **Banff** | **Molecular microscope** | | | | |
| --- | --- | --- | --- | --- | --- | --- | --- |
|  |  |  | **Molecular rejection classifier (normal < 0.3)** | **g > 0 probability (normal < 0.28)** | **ptc > 0 probability (normal < 0.24)** | **Main rejection phenotype** | **Sign-out** |
| 1 | 12m | g1, cg1, ptc1, C4d2 | 0.52 | 0.69 | 0.55 | 86% EABMR | Moderate early-stage ABMR |
| 5 | 8d | v3, TMA, g0, ptc2 | - | - | - | - | - |
| 5 | 12m | ti1, i-IFTA1, t-IFTA2 | 0.42 | 0.6 | 0.59 | 79% EABMR | Moderate early-stage ABMR |
| 10 | 6d | TMA, kidney infarction | - | - | - | - | - |
| 14 | 12m | g1, cg0, ptc1, C4d0 | 0.48 | 0.32 | 0.56 | 57% EABMR | Mild early-stage ABMR |

# **Supplementary Table 5.** Development of donor-specific antibodies of each enrolled patient during the 12-month study period

| **Patient ID** | **Group** | **Primary outcome** | **Molecular rejection** | **Time to event** | **HLA antigen** | **DSA MFI** | | | |
| --- | --- | --- | --- | --- | --- | --- | --- | --- | --- |
|  |  |  |  |  |  | **Pre-Tx** | **M3** | **M6** | **M12** |
| 1 | IVIG- | Y | Y | 12m | DQ5 | 5097 | 16364 | 10568 | 3617 |
| 2 | IVIG+ | N | N | - | DQ7 | 7203 | 6112 | 4816 | 3814 |
| 3 | IVIG- | N | N | - | DQ7 | 6180 | 0 | 0 | 0 |
| 4 | IVIG+ | N | N | - | A2 | 2317 | 1556 | 2555 | 2482 |
| 5 | IVIG- | Y | Y | 8d | A2 | 1389 | 0 | 0 | 0 |
|  |  |  |  |  | Cw12 | 5067 | 2580 | 3533 | 2938 |
| 6 | IVIG- | N | N | - | A2 | 1402 | 1148 | N/A | 0 |
| 7 | IVIG+ | N | N/A | - | DQ5 | 2111 | 0 | 0 | 0 |
| 8 | IVIG+ | N | N | - | A3 | 1139 | 0 | 0 | 0 |
| 9 | IVIG+ | N | N | - | DQ6 | 4809 | 0 | 0 | 0 |
| 10 | IVIG- | Y | N/A | 6d | DQ2 | 5166 | graft failure | | |
| 11 | IVIG+ | N | N/A | - | Cw7 | 2530 | 0 | 0 | 0 |
|  |  |  |  |  | DQ7 | 3126 | 0 | 2165 | 0 |
|  |  |  |  |  | DR52 | 2561 | 0 | 0 | 0 |
| 12 | IVIG- | N | N/A | - | B8 | 1504 | 0 | 2469 | 3419 |
|  |  |  |  |  | B27 | 1338 | 0 | 0 | 0 |
| 13 | IVIG- | N | N/A | - | A25 | 3520 | 0 | 0 | 0 |
| 14 | IVIG+ | N | Y | 12m | cw9 | 2369 | 0 | 0 | 0 |
|  |  |  |  |  | DQ6 | 2991 | 0 | 0 | 0 |
| 15 | IVIG+ | N | N | - | DQ2 | 3250 | 3247 | 3178 | 0 |
|  |  |  |  |  | DR52 | 2004 | 2396 | 0 | 4131 |
| 16 | IVIG+ | N | N | - | DQ2 | 2636 | 0 | 0 | 0 |
| 17 | IVIG+ | N | N | - | A11 | 0 | 2789 | 0 | 0 |
|  |  |  |  |  | B52 | 2303 | 0 | 0 | 0 |

**Abbreviations:** Y – yes, event occured; N – no, event did not occur; N/A - molecular assessment not available

# **Supplementary Table 6.** Therapeutic drug monitoring

|  | **IVIG+ (n = 10)** | **IVIG- (n = 7)** | **p-value** |
| --- | --- | --- | --- |
| rATG cumulative dose, median (IQR) | 6.3 (5.9 – 6.8) | 6 (5.2 – 6.8) | 0.6 |
| tacrolimus through level at week 1, median (IQR) | 15.1 (11.5 – 16.6) | 10.8 (8.3 – 12.2) | 0.07 |
| tacrolimus through level at month 1, median (IQR) | 8.6 (7.2 – 9.6) | 10 (7.9 – 11.4) | 0.59 |
| tacrolimus through level at month 3, median (IQR) | 8.5 (7.4 – 10.1) | 8.6 (7.5 – 10.3) | 1 |
| tacrolimus through level at month 6, median (IQR) | 6.2 (5.1 – 7.4) | 8 (6.8 – 10.1) | 0.18 |
| tacrolimus through level at month 12, median (IQR) | 7.2 (7 – 9.2) | 8 (6.3 – 9.2) | 0.77 |

Abbreviations: C_0_: through level; CNI: calcineurin inhibitor

# **Supplementary Material 1.** Statistical analysis plan

**Description of Statistical Methods**

Primary hypothesis, that rATG/IVIG induction protocol is as effective as rATG alone in defined cohort of HLA incompatible kidney transplant recipients, will be tested in non-inferiority design by one-sided 90% confidence interval, which will be calculated for the difference in proportions between the groups. Also, the standard two-sided 95% confidence interval will be constructed for the main outcome. Primary objective is the combined endpoint defined as biopsy proven antibody-mediated changes and/or TCMR regardless the biopsy indication in HLAi kidney transplantation up to 12 months post-transplantation. Null hypothesis is that perioperative desensitization using rATG alone has higher efficacy failure in composite endpoint defined as biopsy proven antibody-mediated changes (Banff 2017, Category 2) and/or TCMR (Banff 2017, Category 4) in HLAi kidney transplantation compared to rATG along with IVIG.

If the upper limit of the confidence interval for the difference pr^ATG^-pr^ATG+IVIG^ exceeds 20% (non-inferiority margin), we will not reject the null hypothesis about the inferiority of rATG alone compared to rATG/IVIG. If the upper limit of this confidence interval is smaller than 20% (non-inferiority margin), then the null hypothesis is rejected in favour of the alternative hypothesis, which means that that the rATG treatment is non-inferior to rATG/IVIG treatment.

Descriptive statistics will be used for both primary and secondary outcomes. Continuous secondary outcomes will be summarized by mean, standard deviation, median, interquartile range, minimum and maximum and categorial outcomes will be presented as proportions in each category. Survival outcomes will be examined in Kaplan-Meier plots.

Differences in secondary outcomes of the two treatment groups will be tested by two-sided 95% confidence intervals. Differences between category outcomes will be tested by Barnard’s test since there is only a single fixed marginal (the number of patients in the study groups) in the trial, for continuous variables the non-parametric Wicoxon test will be used. In the case of survival analysis, differences will be tested by log-rank test.

Next, secondary objectives will be evaluated. Differences in incidence of chronic active ABMR, acute TCMR, and chronic active TCMR will be tested after 12 months post-transplant. eGFR and ACR are measured as continuous variables. Differences in mean/median between treatment groups will be tested in three time points separately at visit M3, M6 and M12. Differences in DSA and de novo DSA be tested separately at visit M3, M6 and M12. Mortality, graft loss, metabolic, malignant and cardiovascular co-morbidities, incidences of BKV, CMV and EBV replications detected by PCR, incidence of viral and bacterial complications and incidence of study treatment discontinuation will be assessed cumulatively for all 12 months post-transplantation.

**The Number of Participants**

The sample size was determined based on the null hypothesis about non-inferior incidence of efficacy failure composite endpoint in group of patients using rATG alone in comparison with group of patients using both rATG and IVIGs. Based on local experience with these medical events we assume the incidence of composite endpoint in combined treatment group to be 45 %. Non-inferiority margin is based on clinical experience and is set to 20 %. There is no reliable evidence from clinical studies for rejection incidence in patient group defined in this protocol. The Czech Republic is a specific region due to a higher rate of re-transplantations in comparison to other countries, and due to transplantations performed in patients with a worse match. Thus, the results of international studies cannot be extrapolated to the Czech population. According to the centre's experience, the incidence rates of ABMR varies between 6% to 40% between low-risk and high-risk patients. The incidence of TCMR varies between 5% to 30%. The sample size was calculated by the one-sided chi2 test for two independent groups with the significance level of 10 %, test power 80 %. The sample size is calculated on 110 patients (55 patients for the treatment arm). When counting a 20 % dropout, the sample size is 138 patients (69 patients for the treatment arm).

**The Level of Statistical Significance**

For testing the primary hypothesis, a one-sided 90% confidence interval will be used (non-inferiority design with margin of 20 %). Also, the standard two-sided 95% confidence interval will be constructed for the main outcome. For the secondary hypotheses, two-sided 95% confidence intervals will be used and statistical testing will be made on the 5% level of statistical significance. The original level of statistical significance for the main outcome was chosen because, based on clinical experience, the number of patients in this specific group was expected to be small. Statistical significance on the lower level than 10% would increase the required sample size to such an extent it would simply not be possible to enroll enough patients and it would interfere with the feasibility of the clinical trial. This issue does not apply to the secondary outcome, so the standard 5% significance level will be used.

**Procedure for Accounting for Missing, Unused, and Spurious Data**

The formal-logic control of data will be done at the beginning of statistical analysis for detection of missing numbers, unused or inconsistent data. If statistician finds more than 10% missing, unused or inconsistent data in one variable, it is necessary to contact Investigator for discuss representativeness of this events. Unused or inconsistent data will be classified with missing data together as ‘incorrect or missing data’. The number of incorrect or missing data will be the part of all outcomes from descriptive analysis. Into primary analysis can be involved only patients with correctly filled in variables relevant for assessment of efficacy failure composite endpoint at baseline visit and at visit M12 after 12 months after transplantation.

**Analysis sets**

The basic descriptive statistics of patient demography, analysis of Trial Protocol deviations and causes of patient withdrawal will be done for all patients who will be randomized (intention-to-treat approach). Final analysis and detailed assessment of primary and secondary efficacy endpoints will be done for per-protocol population, defined as all subjects who fulfil the protocol in the terms of the eligibility, interventions, and primary outcome assessment.

**Interim analysis**

One interim analysis is planned. The interim analysis can call for potential termination or appropriate modification in sample size. The interim analysis will be focused on descriptive statistics and will be performed when 50% of patients with completed 6 months follow-up (M6) are enrolled. To ensure that study participants are not exposed to excessive risks, incidence of ABMR in experimental arm (PE/PP + rATG) will be assessed. In case the incidence of ABMR in the experimental arm is higher than 50%, the clinical trial will be terminated.

**Statistical software**

All statistical analyses will be conducted with the latest version of R (R: A language and environment for statistical computing. R Foundation for Statistical Computing, Vienna, Austria. URL [https://www.r-project-org](https://www.r-project-org/))).

# **Supplementary Material 2.** Capsule Sentence Summary

This study aimed to determine non-inferiority of IVIG-sparing regiments in HLA-incompatible kidney transplantation. There were 3 (42.9%) ABMR cases in the IVIG- arm and 0 cases in the IVIG+ (p=0.026). The results, although not definitive, do not support this approach.
